# Supplementary material for: Transcriptomic and Translatomic Analyses Reveal Insights into the Signaling Pathways of the Innate Immune Response in the Spleens of SPF Chickens Infected with Avian Reovirus
Source: Viruses. 2023 Nov 29;15(12):2346. doi: 10.3390/v15122346 (PMC10747248; doi:10.3390/v15122346)
Supplement: Supplementary file 1 [file viruses-15-02346-s001.zip › Supplemental Table 1.docx]

| Sample | Clean_Reads | Q20 | Q30 | Mapped Reads | Mapping Rate |
| --- | --- | --- | --- | --- | --- |
| Ribosomal profiling data | | | | | |
| CON1 | 13,119,430 | 98.32% | 95.22% | 6,257,353 | 47.70% |
| CON2 | 14,840,466 | 98.54% | 95.60% | 7,034,151 | 47.40% |
| ARV1 | 16,299,860 | 98.56% | 95.58% | 8,866,426 | 54.40% |
| ARV2 | 16,063,147 | 98.36% | 95.19% | 7,823,279 | 48.70% |
| RNA-Seq data | | | | | |
| CON1 | 17,359,568 | 97.05% | 92.53% | 8,735,246 | 50.32% |
| CON2 | 17,863,633 | 96.98% | 92.43% | 9,445,466 | 52.88% |
| ARV1 | 14,525,168 | 97.08% | 92.63% | 7,883,538 | 54.28% |
| ARV2 | 16,547,030 | 97.09% | 92.73% | 10,701,817 | 64.68% |

Supplemental Table 1. Statistic on the ribosomal profiling data and RNA-Seq data.
