# Supplementary figures and images for: Transcriptomic and Translatomic Analyses Reveal Insights into the Signaling Pathways of the Innate Immune Response in the Spleens of SPF Chickens Infected with Avian Reovirus
Source: Viruses. 2023 Nov 29;15(12):2346. doi: 10.3390/v15122346 (PMC10747248; doi:10.3390/v15122346)

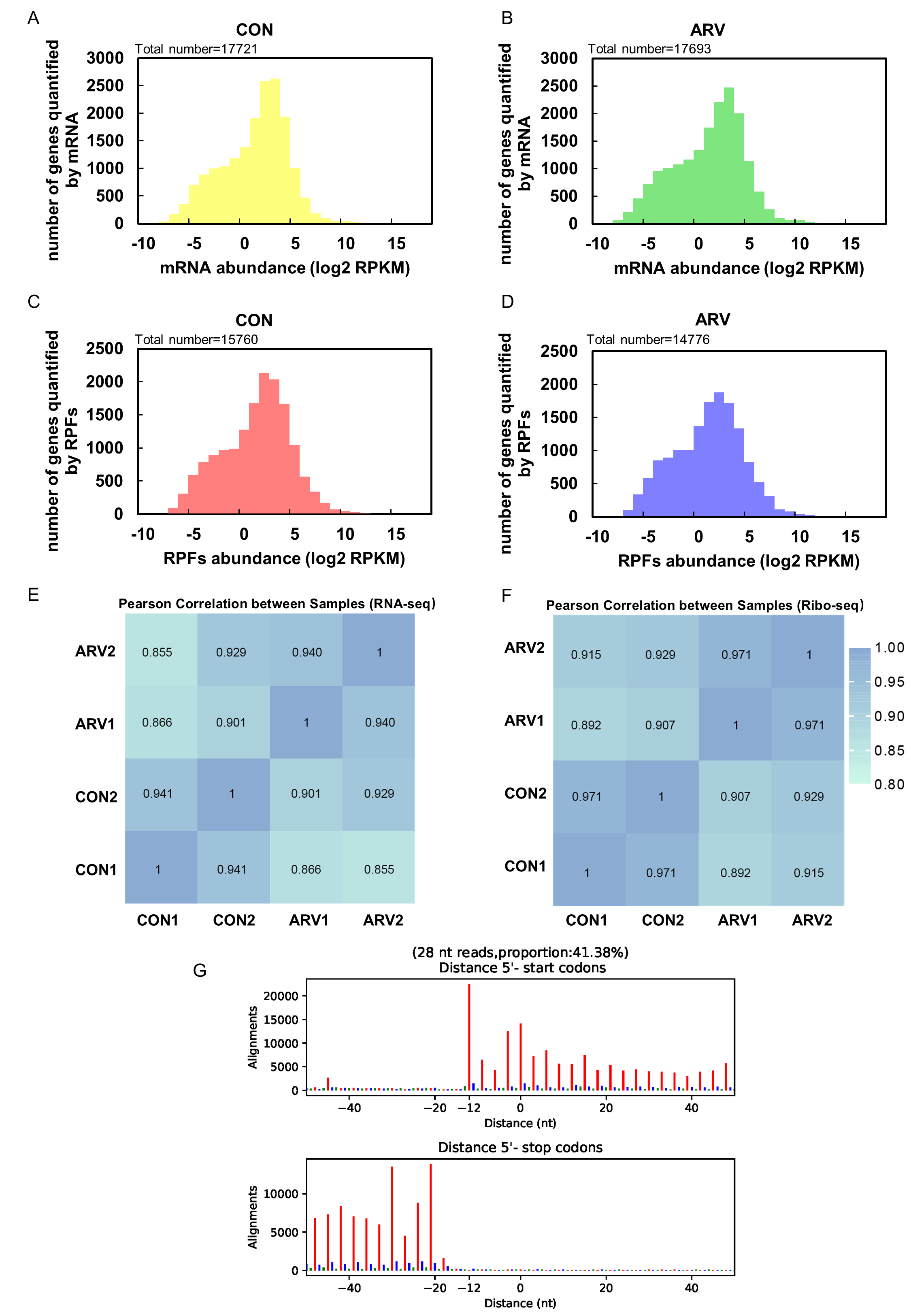

Supplement: Supplementary file 1 [file viruses-15-02346-s001.zip › Supplemental Figure 1.tif]

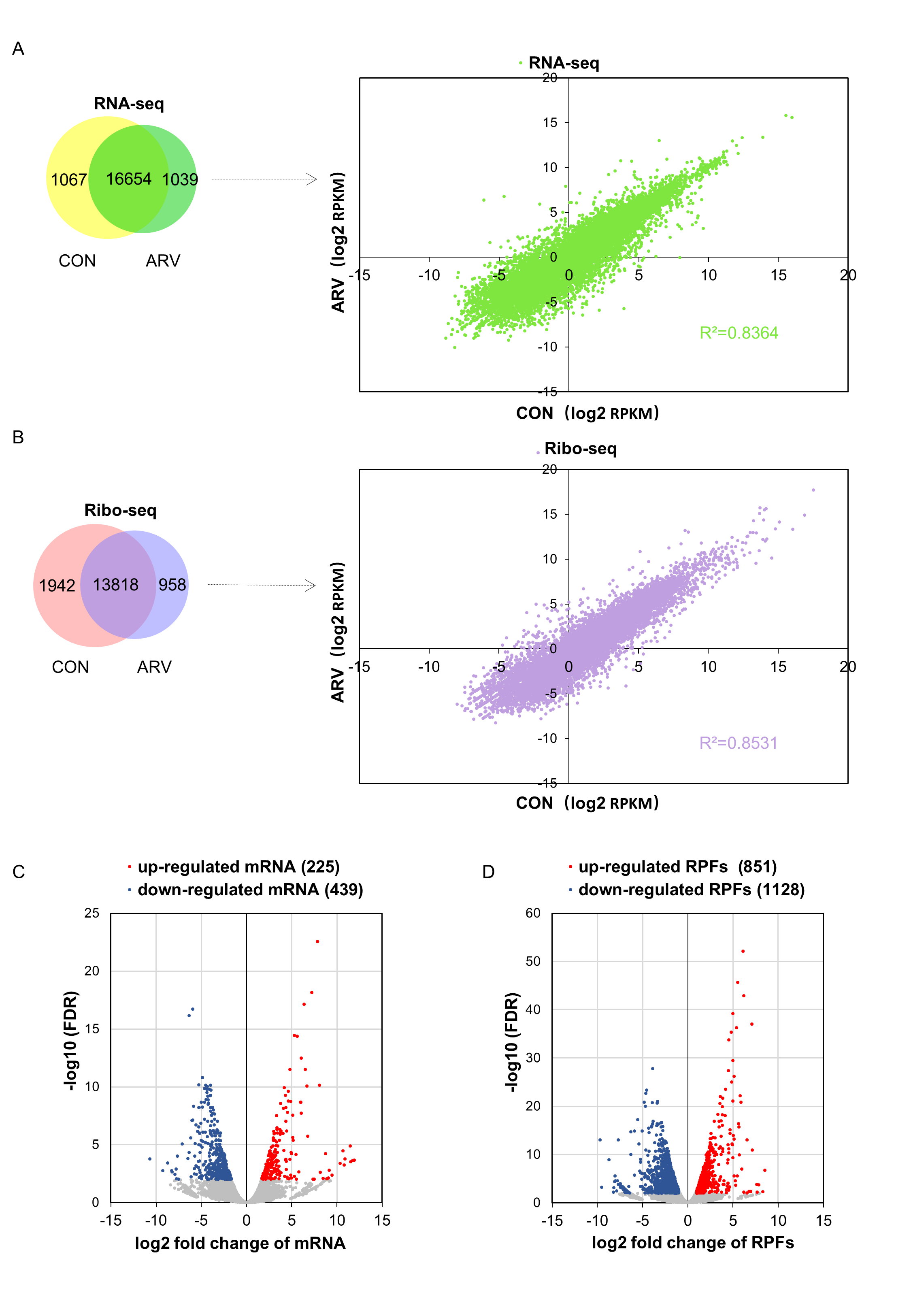

Supplement: Supplementary file 1 [file viruses-15-02346-s001.zip › Supplemental Figure 2.tif]
